# From consultation to participation in public health research: reflections on a community-based research partnership

**DOI:** 10.1186/1756-0500-7-936

**Published:** 2014-12-19

**Authors:** Lauren J Breen, Moira O’Connor

**Affiliations:** School of Psychology and Speech Pathology, Faculty of Health Sciences, Curtin University, GPO Box U1987, Perth, 6845 Western Australia Australia

**Keywords:** Participatory action research, Community-based participatory research, Trauma, Injury, Bereavement, Consumer involvement

## Abstract

**Background:**

Road traffic crashes and their outcomes are substantial global public health issues and public health initiatives are increasingly involving relevant community members in order to create sustainable change. This paper describes an applied research project utilizing participatory methods to establish a road trauma support service in Western Australia and reflects on the extent of participation in the community-based research partnership. Community-based participatory research (CBPR) provided the basis for the research project conducted in partnership with 34 government and non-government agency representatives and people affected personally by road trauma and which resulted in 22 recommendations for establishing the service.

**Findings:**

Attempts to position the group as co-researchers highlighted the dynamic interplay of factors that hinder and enable participation in participatory research. Barriers to participation within the research process included the limited time and funds, reluctance to share authorship, and a lack of clarity regarding roles and processes. Factors that enabled participation were the recognition of each member’s expertise, providing different forms and methods of communication, and the reimbursement of costs according to role.

**Discussion:**

In May 2012, the Government of Western Australia announced it would fund the recommendations and Road Trauma Support Western Australia was launched in November 2013. Notwithstanding this successful outcome, there were varied experiences of participation in the research process, and this was despite the use of a research methodology that is by definition participatory, with explicit and embedded participatory structures and processes. The research project shows that elements of CBPR can be incorporated into public health research, even in projects with externally-imposed time and budget constraints.

## Background

Road traffic crashes result in the deaths of approximately 1.24 million and injuries to 20 to 50 million people every year [1]. Globally, traffic crashes are the eighth leading cause of death and the leading cause of death for people aged 15 to 29 years [2]. In 1974, the World Health Assembly declared traffic crashes to be a substantial public health problem and the increasing scope of the issue prompted the World Health Organization to dedicate its annual World Health Day in 2004 to road safety [3].

Crashes and their consequences affect drivers, riders, and passengers; their family, friends, and colleagues; witnesses and emergency service workers; and members of the wider community. Outcomes include serious physical injuries and temporary or permanent disability [4, 5], intense distress and grief [6, 7], post-trauma reactions and psychiatric disorders [8, 9], social isolation and stigma [10, 11], decreases in quality of life [12], carer burden [13, 14], and considerable financial costs [15, 16]. Notwithstanding the frequency of crash injuries and fatalities and the differential risk borne by vulnerable road user groups [17], the people affected by these outcomes are neglected [3]; this is despite post-crash care being positioned as one of five pillars of road safety, alongside road safety management, safer roads, safer vehicles, and safer road users [1].

Despite these consequences, the focus of traffic crash prevention and management has traditionally been on the ‘hardware’ aspects such as road engineering, vehicle safety, and punitive behavioral measures (e.g., licensing, fines) to shape road user behavior [1, 3]. In contrast, traffic crash consequences are often ‘invisible’ to service providers and the wider community [10, 11]. Furthermore, research has highlighted the disconnect between government rhetoric, which draws on a community discourse to emphasize road safety as a community responsibility, with practices that discourage community narratives from being voiced, by regulating “who is involved, how often they are involved, and the roles of those involved” ([10], p. 47). Over the past few decades, several countries have seen the emergence of grassroots community groups advocating on behalf of traffic victims (e.g., Mothers Against Drunk Driving in the United States; Fédération Européene des Victimes de al Route/ Federation of European Road Traffic Victims). While these groups differ in their origins, resources, and objectives, many were established by people who are injured or bereaved as a result of crashes. Together, these groups organize events for memorial and remembrance purposes (e.g., the annual World Day of Remembrance for Road Traffic Victims), develop and distribute brochures and other materials, many of which contain stories of road traffic victims and their families; campaign for improved road infrastructure and crash prevention; and advocate for improved post-crash responses, with a focus on emergency care as well as social, medical and legal services [18].

It is increasingly recognized that mainstream research methods, which are led and owned by researchers as experts and rarely give any consideration to context, are often ill-suited to the investigation of complex health problems [19]. Alongside this, there has been an increasing awareness of the importance of communities being actively engaged in research to identify, define, and discover ways to resolve or solve the health problems affecting them [20–22]. These developments underpin the increasing use of participatory research methods that promote the involvement relevant community members in order to address health issues, resolve disparities, and create sustainable change [23, 24].

Community-based participatory research (CBPR) is an overarching term used to describe research a collection of research approaches variously described with monikers such as action research, participatory action research, and cooperative inquiry [25]. There are three defining features of CBPR – the research occurs *with* exploited/oppressed communities rather than *on* them [26]; the research addresses the specific concerns of a community to achieve change, often in relation to health disparities [27–30]; and the research involves a research and action process [24]. The co-researchers are typically involved in decisions about all stages of the research [31]. However, CBPR research has its own challenges, notably the extent to which the process is participatory. Typically, explicit accounts of initiating and sustaining participation are not described in published papers and the challenges of promoting participation tend to be oversimplified or obscured [32, 33].

## Methods

This project emerged from an identified need in the community. A small group of people bereaved through traffic crashes had been active in advocating for a road trauma support service in Western Australia since 2000 but, until recently, their campaign for a structured support service for people affected by road trauma had not been successful (see [10], for a discussion of this action). The absence of a dedicated road trauma service was especially problematic, given that Western Australia consistently demonstrates the highest traffic crash fatality rate of all Australian states [34]. Further, there are considerable gaps and limitations in the current services available in Western Australia, which are difficult to identify, costly to access, limited due to time restrictions or staffing resources, and available only in certain regions rather than state-wide.

Due to these gaps, in mid-2010, the Western Australian Department of Health commissioned expressions of interest from researchers to investigate mechanisms and associated costs and to make recommendations in regard to establishing a road trauma support service in Western Australia to provide sustainable peer support and professional counseling for road trauma victims, family members, witnesses and others who are adversely affected by road trauma events. Although the timeline and general aims were established by the Department of Health, there was greater leeway in refining the aims are determining the ways in which they would be addressed. In proposing our research plan (which was ultimately funded), we drew upon CBPR to privilege the perspectives of people working with, or affected by, road trauma with a goal of producing health service change.

Following approval from the Curtin University Human Research Ethics Committee (approval number HR29/2011; in compliance with the Helsinki Declaration), a stakeholder reference group was formed, comprising 34 representatives from government (e.g., police, hospitals, victim support, emergency services, coroner’s office) and non-government agencies (e.g., carer and disability advocacy, injury control, a road user group, the peak body for support groups), as well as community members bereaved by and/or injured in traffic crashes. Several group members already knew each other due to their ongoing advocacy for road trauma support and/or involvement in previous initiatives (e.g., the development of young driver training policy, the unveiling of a crash fatality remembrance memorial). Face-to-face meetings were complemented by regular, ongoing contact via email, post, and telephone.

The group decided to examine existing road trauma support programs in Australia in order to determine the parameters of a similar service in Western Australia and assisted in the development of a semi-structured interview protocol. Using this protocol, we conducted telephone interviews with key personnel at each service and these were complemented by the analysis of each service’s websites, mission statements, annual reports, brochures, and evaluations. Attention was paid to the history and development of each service; promotion of services to service users and referring professionals; referral pathways to and from each service; fees; scope and duration of service delivery; recruitment, training, and appointment of paid and volunteer staff; resources required, including accommodation, administration, computing, communications and promotional materials; metropolitan and regional service delivery; establishment and ongoing annual costs and funding sources; and service evaluation. We wrote case study summaries of the services.

The group used the summaries to determine which services would be visited by the first author (the project’s budget would not cover visits to all services or the travel of more than one person), and finalize the data collection strategy. Three services – those in the states of Victoria, South Australia, and Tasmania – were chosen for visits because they were considered to offer the most comprehensive road trauma services. Using the protocol described earlier, the first author conducted semi-structured, face-to-face interviews of approximately one hour each with key staff members (i.e., counselors, managers, executive officers, volunteer coordinators) from the three services in order to expand on the information gleaned from the case studies. Additionally, the travel to each service facilitated observations of each service’s location and resources (e.g., office space, number of staff, access via public transport, and parking). This analysis of existing services elsewhere in Australia revealed each service’s strengths and limitations, which informed the recommendations for establishing the road trauma support service in Western Australia.

We wrote a draft report and disseminated it within the group for comments and changes and made every effort to incorporate them into the final report. Once complete, the final report [35] was made available (in hard and electronic copy) to each group member, the visited services, and to the Department of Health. The report included 22 recommendations for the establishment of the road trauma support service. The findings and recommendations have been published previously [36]. What follows is an overview of the research process and the lessons learned in attempting to optimize the participation of government and non-government agency representatives and community members affected by road trauma in the research process.

## Findings

In conducting this research, the tension between the ideal and reality of participation became apparent. Ultimately, this tension was reinforced by power differentials between the researchers and the group, and between group members, and several challenges arose as a result. These challenges encountered in attempting to optimize participation, as well as the facilitators of participation, are discussed below. Articulating these challenges allowed us to implement strategies to mitigate their impact, leading to an overall successful outcome.

### Challenges to participation

First, the development of co-researcher partnerships between group members and between the university researchers and co-researchers was limited by time and budgetary constraints. Our ability to develop a true partnership with the group while completing the contracted objectives of the project was complicated by the tight timeline required by the funding body. We negotiated a three-month extension to the project, and while this extension helped, the process adopted did not engender full participation from the outset. Instead, it began as a consultative process and developed in its participatory nature over time, a common distinction in participatory research [37]. Additional time would have allowed more discussion about the scope of the study, allowing the group to provide further input. Inclement weather prevented the majority of group members from being present at one face-to-face meeting, meaning that the majority of members were not there to participate in the decision-making discussion. Unfortunately, the project’s timeline also precluded rescheduling the meeting and the authors communicated via email and telephone with group members who were not able to attend. Additionally, we would have preferred the group to nominate a member to accompany the first author on the visits to existing services elsewhere in Australia. Such visits would have been invaluable to the service providers and to the community members as well as an indication of the co-researcher status we were attempting to foster. However, this was not possible due to the budget limitations.

Second, surprisingly, we encountered hesitancy to share authorship among group members. One group member expressed discomfort concerning sections of the draft report that implied the recommendations were supported by the group members’ organizations (where applicable). This member explained that the report would need to be endorsed through each organization’s board of management for the report to state or imply as such. This perspective was opposed by other group members who expressed disappointment and anger and inferred that the particular organization was against the proposed funding model from the beginning. However, the time restrictions on the project meant that we would be unable to gather the various permissions for everyone to author the report so we decided that the authorship had to be separated from participation in the group. Duckett and Fryer [27] wrote about separating the research process from the publication, so they authored publications without the co-researchers because the co-researchers wished to remain anonymous. We reworded relevant sections of the draft and asked the group about their suggestions for alternate wording and confirmed their satisfaction with the changes. One member emailed that she was satisfied with the changes and appreciated the participatory approach taken to getting feedback on the draft:

It is difficult to manage a large and diverse group under any circumstance let alone when it involves a highly emotional and personally relevant topic. I am grateful of your consideration of my feedback and appreciate that you have accommodated that.

Third, despite our attempts to communicate clearly, at times there appeared to be a lack of understanding about the processes necessary in research. Ultimately, the Department of Health was the client, which weakened our attempts to promote the group’s ownership of the study. For example, some group members expressed frustration at the time it took to complete the project and informed a member of the opposing political party. This politician emailed an author to inquire about the progress of the study and the anticipated completion date. The author replied by suggesting that the member contact the Department of Health and/or the Minister for Health. However, the politician and some members of the stakeholder reference group described the reply as being obstructive as they did not understand that ethically it was not appropriate to provide information to third parties.

As further example, the university’s public relations section organized a media campaign wherein articles relating to the research appeared on the university homepage, a science network website, and in various newspapers. We asked that the group, or at least some members, be featured in the newspaper articles and accompanying photographs but it became clear that the media interest was based on a university-generated press release and the newspapers’ journalists stated they were not interested in featuring group members. Notwithstanding these issues, it was serendipitous that there was little conflict because, given the time constraints, we did not develop a group protocol or terms of reference document. Clarity concerning role and processes is typically essential in outlining the purpose and objectives of the research project and the membership and governance of the group [19].

### Facilitators of participation

In order to promote participation and mitigate tokenism, we first aimed to include an approximately equal number of representatives from services and people with personal experiences of traffic crash consequences and, throughout the project, we described both types of representatives as experts – one with expertise in service delivery and the other with expertise from personal experience. The inclusion of the community members meant that the issues under discussion were related to ‘real-life’ experiences, situations, and realities rather than abstractions [38]. For instance, in the third meeting, a service representative suggested that current services were adequate and there was no need for a comprehensive road trauma support service. Three community representatives (one who had been injured and two who were bereaved) each shared some of their experiences of attempting to negotiate services, which highlighted the ‘real-world’ gaps in the current system. Additionally, in the report, we acknowledged each member by name in alphabetical order and without distinction between the service and community representatives.

Second, we were acutely aware of the differences in the ability of individual group members to participate in the research and used several methods of communication to mitigate these differences. For instance, while the service representatives had ready access to email, fax, and printing facilities, this was sometimes not the case for the community representatives. In recognition of these differences, all group members had the option of receiving information via email or via post. Many community representatives requested that documents were posted as they did not have facilities to print them. Radermacher and Sonn [39] described the different strategies they used in disseminating information to different group members to maximize access and participation. However, they reported that their attempts to use different methods of communication still had a deleterious effect on the ability of some members to participate fully.

Third, we were aware that service representatives were paid by their employers for their participation while the community representatives were representing themselves in an unpaid capacity. As such, the members with personal experiences of traffic crash consequences each received a shopping voucher in recognition of their time and associated expenses such as transport and parking. Together, we believe these strategies lessened the impact of the challenges. Although we did not formally gather feedback from the group (e.g., through an anonymous questionnaire) on their perceptions of participation throughout the research process, spontaneous feedback from group members provide some evidence of support for our reflections. One member emailed:

We value the work carried out by the dedicated investigators who demonstrated both compassion and professionalism throughout the research process… Importantly, the project was based on the contributions from the individuals and families who have campaigned for many years for a road trauma support service in Western Australia. The project has also taken into account key human rights principles of self determination, partnerships in research and inclusion within society.

## Discussion

The destabilization of power hierarchies and the creation and maintenance of equitable power structures are major challenges of CBPR and were challenges we faced at times throughout the study. Typically, discussions about unequal power focus on the difference between academic researchers and the co-researchers [31, 32]. In this study, however, the differences between the community members and the service representatives were just as prominent as those between the group and the researchers. These tensions were acknowledged and sometimes compromises were made. Despite attempts to include the stakeholder reference group as equal co-researchers, the process, at least at first, would best be described as consultative. However, over time, the process became increasingly participatory.

The progression from participants to co-researchers was evident in the group members’ comments on the draft report. Despite the length of the draft (approximately 32,000 words) and the deadline for comments (about three weeks), all members of the group provided thoughtful and constructive comments on it and much discussion emerged from the deep and considered critique of the content. For example, group members (service and community representatives) together expressed concern about a section of the draft where grief had been (inadvertently) described in a medicalized and pathologized manner without adequate contextualization or regard to alternative models and approaches. These members did not hesitate to express their disapproval and provide alternate wording. Other examples included the group members’ questions over the proposed funding model for the service, requests that the report include more detail on the effects of traffic crash injuries and the out-of-pocket costs that are borne by families following traffic crashes, suggestions regarding terminology (e.g., that ‘carer’ or ‘unpaid family caregiver’ were preferable to ‘caregiver’) and formatting (e.g., the use of a table to summarize the strengths and limitations of road trauma support services elsewhere in Australia), and the incorporation of references to state and national road safety strategies.

We believe this progression from consultation to participation was because the members’ participation was meaningful and they felt supported, trusted, valued [40]. While the reference group did not control or direct all aspects of the study, the group had considerable influence over shaping the agenda of the study, the methods used, and the recommendations that were proposed. Ultimately, the participation achieved was extremely positive and bodes well for future research, given the challenges described earlier.

To our knowledge, this is the first project to draw upon CBPR in road trauma research. The use of CBPR in this project demonstrated some of the strengths and challenges of the methodology in developing a community-based research partnership. The development of this partnership facilitated the creation of a space for people with different experiences, opinions, and backgrounds to come together [38], because of their shared interests in road trauma issues, and to effect change. To date, the community-based research partnership has resulted in a change in government policy and practice, with the Government of Western Australia announcing in May 2012 it would fund a service according to the recommendations outlined in the report. The service, Road Trauma Support Western Australia, was launched in November 2013 on the World Day of Remembrance for Road Traffic Victims. Many of the project’s group members are members of the service’s steering group and this is indicative of the way in which the group has continued to have ownership of the project beyond the research phase. The diversity and depth of the stakeholder reference group – its members including representatives from relevant services as well as people affected personally by road trauma – enhances the study’s ability to contribute to policy and practice [41]. Further, the report will provide the basis for the ongoing development and evaluation of the newly-established service.

## Conclusions

Although it is increasingly common for stakeholders to have a role in decisions that affect them, the involvement can often be tokenistic [37] and the action aims tend to be are easier to achieve than the emancipatory aims [42]. Indeed, participatory approaches can be paradoxically used (and abused) to silence debate and defuse conflict between the powerful and powerless [43]. For instance, in health research, there is often one token consumer or one articulate consumer, leading to major power differentials between them and the recognized ‘experts’ in the committee, group, or service [44, 45]. The research project shows that elements of CBPR can be incorporated into public health research, even in projects with externally-imposed time and budget constraints. Clearly, it is the ways in which the participation is facilitated that matters.

In describing a participatory research project that resulted in changes to health policy and practice, this paper contributes to the current discussion concerning the extent of participation in participatory research aiming to address public health concerns. Often, a critical analysis of participation within the research process is glossed over in describing research outcomes whereas in this paper there is an emphasis on the complexities, limitations, and possibilities of participatory research. We highlighted the dynamic interplay of factors that hinder and enable participation in participatory research. These reflections are useful in determining the strengths and limitations of this particular study and may also be useful to others interested in using CBPR for public health outcomes. A key advantage of this design was that it afforded the stakeholders the opportunity to generate meaningful knowledge and sustained action for their community. As a methodology at the intersection of science and practice, CBPR is likely to be an important tool for an action-oriented and community-driven public health.
